# Hierarchical palladium catalyst for highly active and stable water oxidation in acidic media

**DOI:** 10.1093/nsr/nwac108

**Published:** 2022-06-09

**Authors:** Jing Peng, Haofeng Sun, Kun Ni, Jiajing Wu, Xinyu Sun, Yueqi Su, Han Cheng, Yuhua Liu, Yuqiao Guo, Wentuan Bi, Yanwu Zhu, Changzheng Wu, Yi Xie

**Affiliations:** Hefei National Laboratory for Physical Science at the Microscale, CAS Center for Excellence in Nanoscience, iChEM (Collaborative Innovation Center of Chemistry for Energy Materials), and CAS Key Laboratory of Mechanical Behavior and Design of Materials, University of Science and Technology of China, Hefei 230026, China; Hefei National Laboratory for Physical Science at the Microscale, CAS Center for Excellence in Nanoscience, iChEM (Collaborative Innovation Center of Chemistry for Energy Materials), and CAS Key Laboratory of Mechanical Behavior and Design of Materials, University of Science and Technology of China, Hefei 230026, China; Hefei National Laboratory for Physical Science at the Microscale, CAS Center for Excellence in Nanoscience, iChEM (Collaborative Innovation Center of Chemistry for Energy Materials), and CAS Key Laboratory of Mechanical Behavior and Design of Materials, University of Science and Technology of China, Hefei 230026, China; Hefei National Laboratory for Physical Science at the Microscale, CAS Center for Excellence in Nanoscience, iChEM (Collaborative Innovation Center of Chemistry for Energy Materials), and CAS Key Laboratory of Mechanical Behavior and Design of Materials, University of Science and Technology of China, Hefei 230026, China; Hefei National Laboratory for Physical Science at the Microscale, CAS Center for Excellence in Nanoscience, iChEM (Collaborative Innovation Center of Chemistry for Energy Materials), and CAS Key Laboratory of Mechanical Behavior and Design of Materials, University of Science and Technology of China, Hefei 230026, China; Hefei National Laboratory for Physical Science at the Microscale, CAS Center for Excellence in Nanoscience, iChEM (Collaborative Innovation Center of Chemistry for Energy Materials), and CAS Key Laboratory of Mechanical Behavior and Design of Materials, University of Science and Technology of China, Hefei 230026, China; Hefei National Laboratory for Physical Science at the Microscale, CAS Center for Excellence in Nanoscience, iChEM (Collaborative Innovation Center of Chemistry for Energy Materials), and CAS Key Laboratory of Mechanical Behavior and Design of Materials, University of Science and Technology of China, Hefei 230026, China; Hefei National Laboratory for Physical Science at the Microscale, CAS Center for Excellence in Nanoscience, iChEM (Collaborative Innovation Center of Chemistry for Energy Materials), and CAS Key Laboratory of Mechanical Behavior and Design of Materials, University of Science and Technology of China, Hefei 230026, China; Hefei National Laboratory for Physical Science at the Microscale, CAS Center for Excellence in Nanoscience, iChEM (Collaborative Innovation Center of Chemistry for Energy Materials), and CAS Key Laboratory of Mechanical Behavior and Design of Materials, University of Science and Technology of China, Hefei 230026, China; Hefei National Laboratory for Physical Science at the Microscale, CAS Center for Excellence in Nanoscience, iChEM (Collaborative Innovation Center of Chemistry for Energy Materials), and CAS Key Laboratory of Mechanical Behavior and Design of Materials, University of Science and Technology of China, Hefei 230026, China; Institute of Energy, Hefei Comprehensive National Science Center, Hefei 230026, China; Hefei National Laboratory for Physical Science at the Microscale, CAS Center for Excellence in Nanoscience, iChEM (Collaborative Innovation Center of Chemistry for Energy Materials), and CAS Key Laboratory of Mechanical Behavior and Design of Materials, University of Science and Technology of China, Hefei 230026, China; Hefei National Laboratory for Physical Science at the Microscale, CAS Center for Excellence in Nanoscience, iChEM (Collaborative Innovation Center of Chemistry for Energy Materials), and CAS Key Laboratory of Mechanical Behavior and Design of Materials, University of Science and Technology of China, Hefei 230026, China; Institute of Energy, Hefei Comprehensive National Science Center, Hefei 230026, China; Hefei National Laboratory for Physical Science at the Microscale, CAS Center for Excellence in Nanoscience, iChEM (Collaborative Innovation Center of Chemistry for Energy Materials), and CAS Key Laboratory of Mechanical Behavior and Design of Materials, University of Science and Technology of China, Hefei 230026, China; Institute of Energy, Hefei Comprehensive National Science Center, Hefei 230026, China

**Keywords:** palladium, oxygen evolution reaction, acidic condition, strain, electrolysis

## Abstract

Acidic water electrolysis is of great importance for boosting the development of renewable energy. However, it severely suffers from the trade-off between high activity and long lifespan for oxygen evolution catalysts on the anode side. This is because the sluggish kinetics of oxygen evolution reaction necessitates the application of a high overpotential to achieve considerable current, which inevitably drives the catalysts far away from their thermodynamic equilibrium states. Here we demonstrate a new oxygen evolution model catalyst-hierarchical palladium (Pd) whose performance even surpasses the benchmark Ir- and Ru-based materials. The Pd catalyst displays an ultralow overpotential (196 mV), excellent durability and mitigated degradation (66 μV h^−1^) at 10 mA cm^−2^ in 1 M HClO_4_. Tensile strain on Pd (111) facets weakens the binding of oxygen species on electrochemical etching-derived hierarchical Pd and thereby leads to two orders of magnitudes of enhancement of mass activity in comparison to the parent Pd bulk materials. Furthermore, the Pd catalyst displays the bifunctional catalytic properties for both oxygen and hydrogen evolutions and can deliver a current density of 2 A cm^–2^ at a low cell voltage of 1.771 V when fabricated into polymer electrolyte membrane electrolyser.

## Teaser

Hierarchical Palladium is a new model catalyst with both high activity and stability for acidic water oxidation, which can be an ideal alternative to iridium- and rutheniumbased materials.

## INTRODUCTION

Water electrolysis that enables effective utilization of intermittent electricity by its conversion into storable chemical energy greatly boosts the development of renewable energy [1–6]. Compared with alkaline conditions, acidic electrolysis is more preferable in the current stage due to its advantages over current density, voltage efficiency and gas purity [7–12]. However, the sluggish kinetics of the oxygen evolution reaction (OER) on the anode results in large overpotential, which greatly reduces the energy efficiency and hinders the progress of acidic electrolysers [13–15]. Furthermore, the harsh circumstances with strongly corrosive electrolyte and oxidative operation voltages cause significant degradation and decomposition of OER catalysts, rendering only a few noble-metal systems able to survive and be applicable for acidic electrolysis. IrO_2_ and RuO_2_ have served as benchmark catalytic materials in the long term, which can facilitate multiple electron and proton transfer at narrow overpotentials under acidic conditions. Recently, through extending Ir- and Ru-based compounds into different compositions and morphology, an overpotential of <200 mV and stability of dozens of hours can be reached [16–21]. Nevertheless, further improvement requires acidic OER catalysts that offer both high activity and long-term durability so as to satisfy terawatt-scale applications.

Palladium (Pd) is one of the platinum group metals and has been widely used in various catalytic processes including hydrogen evolution [22,23], C–H activation [24,25] and oxygen-related reactions [26–28]. Electrochemical dissolution test indicates that Pd is extremely stable at low pH under steady-state high-potential operation [29,30]. The dissolving rate is only 0.03 ng cm^–2^ s^–1^, several orders of magnitude smaller than Ru and Ir, which promises Pd to be a highly durable acidic OER catalyst. However, due to the overly strong binding of oxygen-related intermediates, it displays poor activity and is seldom used for water electrolysis [26,29,31], whereas if its oxygen-binding energy can be optimized, it is reasonable to obtain an acidic OER catalyst with both high activity and long-term stability.

Herein, we demonstrate a new oxygen evolution catalyst: hierarchical Pd with both high activity and stability. Through thermal electrochemical etching, hierarchical Pd is transformed into nanoparticles anchored upon a porous Pd framework. Prominently, the OER performance of the Pd catalyst is dramatically enhanced and exhibits an ultralow overpotential of 196 mV at 10 mA cm^–2^ and high mass activity of 407.2 A g^–1^ at 1.53 V_RHE_ (RHE, reversible hydrogen electrode) in 1 M HClO_4_ when the size shrinks to ∼2.8 nm. The Pd catalysts also display excellent durability with little degradation (66 μV h^−1^ at 10 mA cm^–2^) for 500 h. Density functional theory (DFT) calculation reveals that the tensile strain in Pd (111) facets is responsible for the performance enhancement via optimizing the oxygen binding. Furthermore, the Pd catalyst displays excellent bifunctional catalytic activities for both oxygen and hydrogen evolutions, leading to a water-splitting current density as high as 2 A cm^–2^ at a low cell voltage of 1.771 V to be achieved when fabricated into a polymer electrolyte membrane (PEM) electrolyser. These results indicate that Pd can be an ideal alternative to Ir-based and Ru-based materials for acidic water electrolysis.

## RESULTS AND DISCUSSION

### Preparation and modulation of hierarchical Pd catalyst

**Figure 1.**
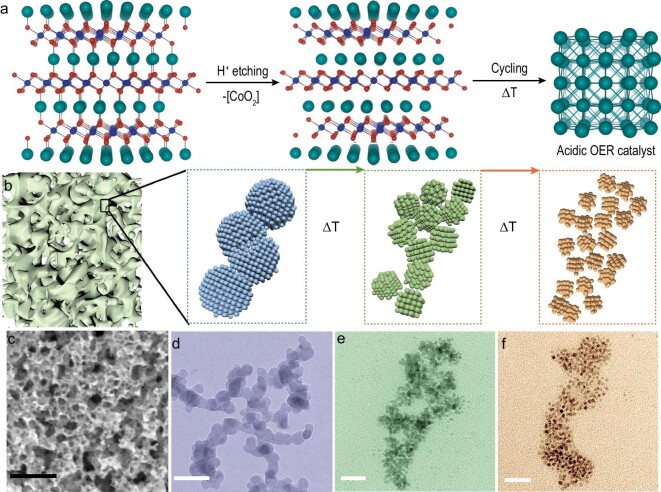
Structure of the hierarchical Pd catalyst. (a) A thermal electrochemical etching strategy was used to remove the [CoO_2_] layers from the PdCoO_2_ lattice in 1 M HClO_4_ under a negative working voltage, forming a Pd catalyst with highly active and stable acidic OER performance. (b) The scheme of a Pd catalyst derived from thermal electrochemical etching with different temperature. (c) SEM image of Pd network. (d–f) TEM images of Pd nanoparticles anchored upon the porous framework with a treated temperature of 25, 40 and 55°C, respectively. The scale bar in (c) is 200 nm and in (d–f) is 20 nm.

The hierarchical Pd catalyst was synthesized through electrochemical etching of layered PdCoO_2_ under acidic medium. The PdCoO_2_ single crystals were immersed and partially etched in 1 M HClO_4_ by means of cyclic voltammetry between 0.05 and –0.35 V for 50 cycles, at which range the hydrogen evolution reaction (HER) can readily take place (Supplementary Fig. S1). During the HER process, [CoO_2_] layers are gradually corroded with the porous Pd framework remaining (Supplementary Figs S2 and S3). The skeleton can shrink to a size of <20 nm in diameter after etching with ultra-small Pd nanoparticles anchored onto it (Supplementary Fig. S4). It is worth noting that the morphology of nanoparticles anchored onto the Pd framework can be tuned by the electrolyte temperature during electrochemical etching (Fig. 1). As the electrolyte's temperature is elevated, the Pd nanoparticles are dissolved into dense and small-sized products. The transmission electron microscopy (TEM) statistic results reveal that the size decreases rapidly and gradually stabilizes to ∼3 nm (Fig. 1f) when heating to above room temperature. The size reduction is presumably ascribed to the hydrogen adsorption in the Pd. Given that Pd can readily adsorb hydrogen and form PdH_x_ at room temperature, a large amount of hydrogen will be adsorbed by the electrochemically etched Pd network during the HER process. As the temperature increases, the soluble hydrogen is released so as to disintegrate large-grained Pd into a small size [32–34].

**Figure 2.**
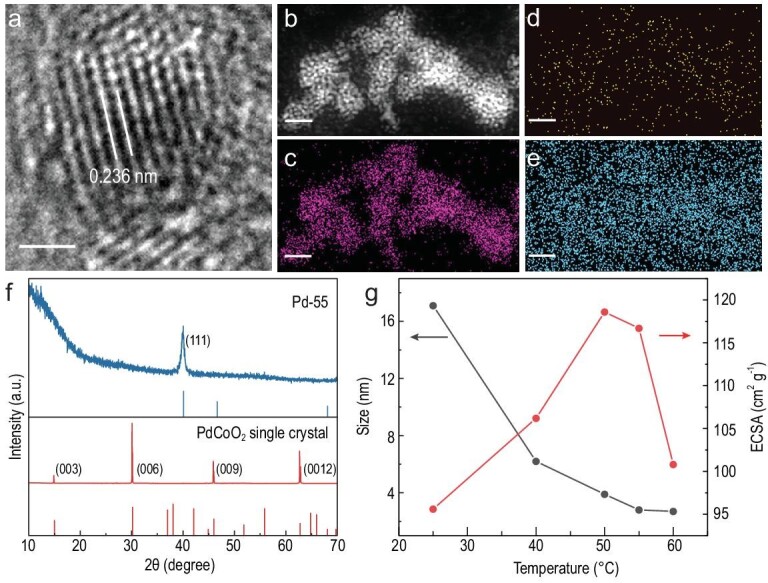
Structural and chemical characterizations of the Pd catalyst. (a) HRTEM of Pd-55. The scale bar is 1 nm. (b–e) HAADF images and corresponding elemental mapping of Pd, Co and O, respectively. The scale bar is 20 nm. (f) XRD pattern of Pd-55 (blue, JCPDS No. 46–1043) and PdCoO_2_ single crystal (red, JCPDS No. 27–1324), respectively. (g) The average size and ECSA of the hierarchical Pd against temperature.

From the high-resolution transmission electron microscopy (HRTEM) image in Fig. 2a, the evident lattice fringes can be observed in 2.8-nm Pd nanoparticles with a spacing distance of 2.36 Å, which is in accordance with the Pd (111) facet. Compared with Pd bulk, the significantly enlarged lattice parameter manifests tensile strain in the Pd nanoparticles after size reduction. High-angle annular dark-field (HAADF) elemental mapping indicates the homogenous distribution of Pd throughout all of the nanoparticles without Co and O residue (Fig. 2b–e). X-ray diffraction and elemental ingredients analysis also reveal the evolution of a corrosion reaction in which the PdCoO_2_ precursor is reduced to elementary Pd (Fig. 2f and Supplementary Fig. S5). As revealed in Fig. 1c, an ultra-high electrochemically active surface area (ECSA) is expected in such a hierarchical Pd structure because of the porous framework, which is beneficial for catalytic reaction. Considering the hydrogen will be incorporated into both the surface and inner of the Pd, the underpotential deposited hydrogen (H_upd_) method will inevitably overrate the ECSA (Supplementary Fig. S6). Therefore, CO stripping was adopted to accurately measure the ECSA of the hierarchical Pd catalyst (Fig. 2g and Supplementary Fig. S7). The ECSA of Pd-25 (25 is the temperature during etching, hereinafter inclusive) can be ≤95 m^2^ g^–1^, indicative of abundant active sites. When the etching temperature increases, the ECSA of the Pd catalyst is slightly enlarged and reaches a peak value of 120 m^2^ g^–1^, which demonstrates that the morphology of the surface Pd nanoparticle makes a limited contribution to the ECSA and catalytic sites, despite the size decreasing rapidly when heated to above room temperature.

### The acidic OER performance of the hierarchical Pd catalyst

**Figure 3.**
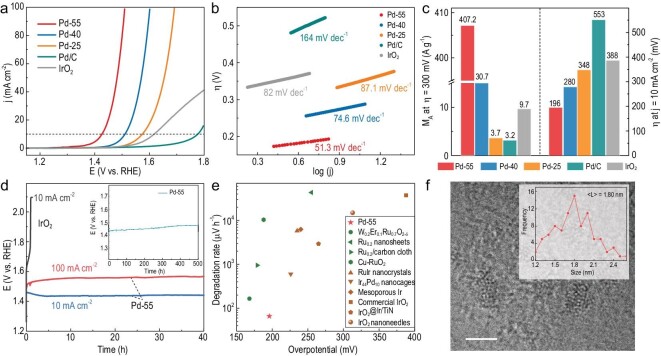
Acidic OER activity and stability of the hierarchical Pd catalyst. (a and b) Linear scan voltammetry curves and corresponding Tafel plots of Pd-55, Pd-40, Pd-25, Pd/C and commercial IrO_2_ in 1 M HClO_4_. (c) Mass activity (left) and overpotential (right) for the catalysts. (d) Chronopotentiometric measurements of Pd-55 and IrO_2_ in 1 M HClO_4_. (e) The comparison of stability and activity in acidic electrolyte for various electrocatalysts [17,18,20,35–40]. (f) HRTEM of Pd-55 after acidic OER test. The scale bar is 2 nm. The inset is the statistic size of Pd nanoparticles.

The OER activities of Pd-25, Pd-40 and Pd-55 were evaluated and compared with commercial Pd/C, IrO_2_ in a conventional three-electrode set-up with 1 M HClO_4_ aqueous solution as the electrolyte. OER polarization curves of the Pd catalyst display enhanced catalytic performances as the etching temperature increases (Fig. 3a and Supplementary Figs S8 and S9). The overpotential at a current density of 10 mA cm^–2^ decreases dramatically from 348 mV for Pd-25 to 280 mV for Pd-40, and Pd-55 displays the best OER catalytic activity with an ultralow overpotential of 196 mV, which surpasses that of commercial IrO_2_ and Pd/C (Fig. 3c). In contrast, the PdCoO_2_ precursors without etching display poor catalytic activity with an overpotential of 528 mV (Supplementary Fig. S9b). Moreover, from Fig. 3b, the Pd catalyst prepared at 55°C exhibits the best kinetics with a Tafel slope of 51.3 mV dec^–1^, which outperforms hierarchical Pd at a lower etching temperature and the benchmark IrO_2_ catalyst. Nyquist plots (Supplementary Fig. S10) studied using electrochemical impedance spectroscopy at 1.5 V_RHE_ show that the charge-transfer resistance decreases from Pd-25 to Pd-55, further manifesting the enhancement of catalytic activity by increasing the etching temperature. Generally, the improvement in catalytic properties can be ascribed to the increment of both active sites and intrinsic activity. To disclose the intrinsic catalytic properties between these samples, currents at 300 mV of overpotential were normalized to the catalyst mass, as shown in Fig. 3c and Supplementary Fig. S11. Pd-25 has a similar mass activity (MA) of 3.7 A g^–1^ to 3.2 A g^–1^ for commercial Pd/C. As the etching temperature increases, the obtained MA of Pd-55 shows >100 times the enhancement and reaches 407.2 A g^–1^, which is far beyond that of IrO_2_. Compared to slightly enriched active sites reflected from ECSA, the considerably enhanced intrinsic activity of Pd nanoparticles dominates the improvement in OER properties as a function of temperature. Many other factors, such as electrochemical cycling numbers or scan rates, were also performed at 55°C to investigate the impacts on OER behavior (Supplementary Fig. S12 and Supplementary Table S1). It can be found that Pd-55 with a smaller particle size has better MA and lower overpotential. The size effect was further evidenced by synthesizing ∼5.4 nm of Pd nanoparticles and obtaining a similar MA of 34.6 A g^–1^ to that of Pd-40 (∼5.8 nm-sized Pd nanoparticles) as revealed in Supplementary Fig. S13. Thus, the distinct improvement in OER performance indicates that the size of the Pd dominates OER intrinsic catalytic activity and Pd-55 that has been shrunk to ∼2.8 nm reveals superior catalytic ability.

Chronopotentiometric measurements were performed to investigate the durability of hierarchical Pd under acidic conditions (Fig. 3d). After 500 h of testing at 10 mA cm^–2^, Pd-55 shows superior stability with little decay in 1 M HClO_4_. When compared to Ir-based and Ru-based catalysts (Fig. 3e and Supplementary Table S2), the Pd-55 degrades at a much slower rate of only 66 μV h^–1^, which demonstrates that Pd can be a promising acidic OER catalyst. Prominently, the mass dissolution rate for Pd-55 was 0.14 ng cm^–2^ s^–1^ during 500-h test. The dissolving rate approaches the bulk counterpart, while the OER activity is improved dramatically. Furthermore, the high durability of >40 h for Pd-55 at 100 mA cm^–2^ also confirms that hierarchical Pd is quite stable under a large current density. Therefore, size reduction can help to promote the catalytic performance of Pd without compromising stability. To further investigate the stability of Pd-55, scanning electron microscopy (SEM) and HRTEM imaging are utilized to analyse its microstructure after OER testing. High porosity is conserved on Pd-55 (Supplementary Fig. S14). Moreover, the Pd nanoparticles maintain good crystallinity and reveal clear lattice fringes after a 500-h test, despite the size significantly decreasing (Fig. 3f). The high crystallinity greatly relieves the dissolution rate of Pd nanoparticles and protects the inner structure, realizing high stability under acidic conditions.

### Theoretical insights on OER reactivity

**Figure 4.**
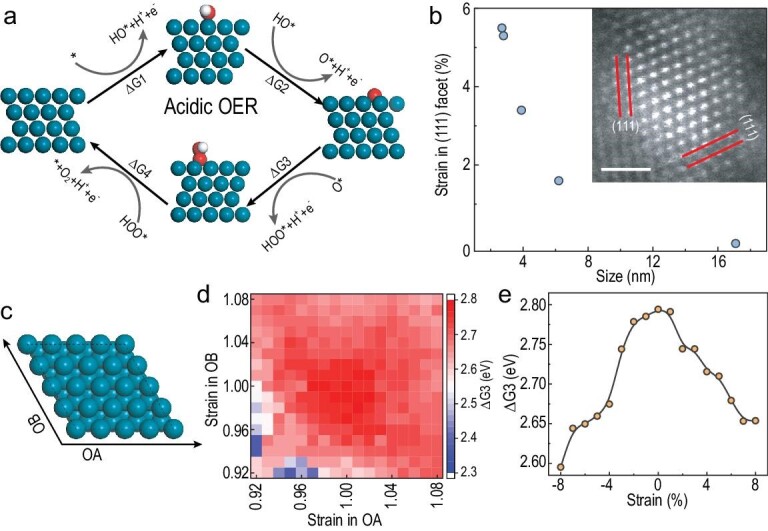
Strain effect for OER performance towards the Pd catalyst. (a) The OER cycle of Pd for the acidic OER process. (b) The lattice strain in Pd nanoparticles with different sizes. The inset is a HAADF-STEM image of a Pd nanoparticle. The scale bar is 1 nm. (c) The (111) facet of Pd along the OA and OB axis. (d) The 2D volcano-type map with respect to strain and ΔG3. (e) The ΔG3 as function of isotropic strain.

DFT calculations were further performed to unravel the origin of enhanced OER catalytic activity stemming from the tuned Pd nanoparticle strain. Four-electron steps (Fig. 4a), which involve three key intermediates (HO*, O* and HOO*), usually dominate the overall activity in the OER process. The corresponding free energies were calculated on the perfect (111) facet. As can be seen in Supplementary Fig. S15, the quite low ΔG2 of only 0.13 eV demonstrates binding of O* that is too strong, which is consistent with previously reported elucidations towards the poor OER performance of Pd. Consequently, the strong oxygen binding results in an ultra-high energy barrier of ΔG3 (2.79 eV, ΔG_HOO*_−ΔG_O*_) despite HOO* having a normal adsorption energy, and the potential determining steps (PDS) for the Pd catalyst are between HOO* and O*. In our case, the Pd (111) facets are indeed exposed on the Pd nanoparticles, as confirmed by HAADF images in Fig. 4b. Intriguingly, the size difference gives rise to great divergence of plane distances in (111) facets. The distance increases significantly as the size shrinks, thereby bringing in evident tensile strain [23,41]. Therefore, the introduction of lattice strain in the (111) facet likely plays an essential role in improving the OER activity of Pd, especially the energy barrier of the PDS. Accordingly, energy landscape calculations were carried out on 2D Pd (111) surfaces with a different strain (from –8% to 8%) along the OA and OB axis (Fig. 4c). A 2D volcano-type map with respect to the strain and ΔG3 (Fig. 4d) is utilized to represent the variation in OER performance. In contrast to perfect Pd (111), ΔG3 obtains a significant decrease with strain applied, whatever the tensile and compressive way. Prominently, ΔG3 is manifestly reduced by 0.11 eV while a 5% tensile strain exists (Fig. 4e, plotted by the variation of ΔG3 as the isotropic strain), which agrees with the significant enhancement of acidic OER activity for Pd-55 with a size of nanoparticles of <3 nm. Therefore, strain engineering exhibits well the rationalization of the OER reactivity in hierarchical Pd comprising different particle sizes. Furthermore, the mapping in Fig. 4d indicates that the effect of strain in (111) facets is anisotropic to the OER activity, which can guide more modulation freedom for the acidic catalyst.

### Water electrolyser testing for the hierarchical Pd catalyst

**Figure 5.**
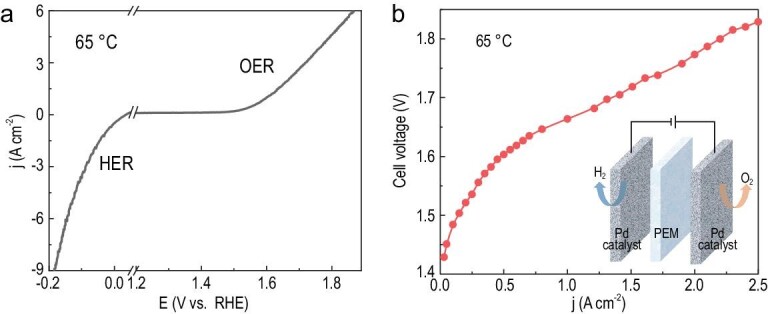
PEM electrolyser performance. (a) The bifunctional catalytic properties of Pd-55 at 65°C. (b) Polarization curves of the electrolyser with Pd-55 (2 mg cm^–2^) as both cathode and anode catalysts.

Owing to the corrosion-resistant porous conductive framework, hierarchical Pd can be an ideal platform to harvest effective mass and charge transport during water electrolysis. The high porosity greatly reduces gas and water percolation limitation when Pd-55 fabricated into a membrane–electrode assembly. Prominently, the Pd catalyst also exhibits superior HER performance under acidic conditions, which obtains a large current density of 3.41 A cm^–2^ under −0.1 V_RHE_ at 65°C (Fig. 5a). A high-performance PEM electrolyser could be fabricated when the bifunctional materials serve as HER and OER catalysts simultaneously. In our acidic water electrolyser, the anode and cathode were both constructed using Pd membranes comprising a Pd-55 catalyst, with mass loading of 2 mg cm^–2^. The fast proton reduction renders the overpotential in the cathode to be neglected and the total overpotential of the electrolyser is mainly contributed from the anode. Thanks to the high acidic OER activity of Pd-55, the overpotential can be significantly reduced. From the polarization curves at 65°C in Fig. 5b and Supplementary Figs S16 and S17, a voltage of only 1.664 and 1.771 V is required to achieve 1 and 2 A cm^–2^, respectively, demonstrating superior performance for acidic water electrolysis [10,42]. Note that further optimizing the structure and configuration of the electrolyser is needed, so as to achieve better performance and upscaling applications.

## CONCLUSION

We reported an electrochemical etching-derived acidic OER catalyst, hierarchical Pd that shows superior catalytic performance with both high activity and stability. The elevated temperature during the etching process can effectively modulate the size of the Pd nanoparticles anchored upon the porous skeleton, thereby improving the OER activities. Prominently, Pd-55 with particle size that has been shrunk to 2.8 nm exhibits optimal performance, where the overpotential at 10 mA cm^–2^ can decrease to 196 mV and the durability reaches up to 500 h in 1 M HClO_4_. High intrinsic activity and DFT calculation indicate that strain effects result in the optimization of oxygen binding with Pd, thereby enabling the inactive Pd to become an excellent acidic OER catalyst that overwhelms IrO_2_. The high performance of the Pd-55 catalyst has been demonstrated in a PEM electrolyser configuration. These results demonstrate that hierarchical Pd can be an ideal alternative to Ir-based and Ru-based materials for acidic water splitting, and also suggest that rational strain modulation and structure design can be powerful strategies for improving the performance of acidic OER catalysts.

## MATERIALS AND METHODS

### Synthesis of PdCoO_2_

The synthesis of millimeter-sized PdCoO_2_ single crystals was carried out according to previous reports in the literature [43]. First, stoichiometric powders of PdCl_2_ and CoO were sealed in an evacuated quartz tube. The tubes were heated to 1000°C in 6 h and then cooled down quickly to 580°C in 1.5 h. The intermediates were heated again to 700°C and kept for 80 h, and finally cooled down to room temperature at a rate of 40°C h^–1^. Products were washed with water and ethanol to remove CoCl_2_, yielding metallic crystalline hexagonal lamellae.

### Sample and electrode preparation

A hierarchical Pd catalyst was prepared through the thermal electrochemical etching process. The large-sized PdCoO_2_ single crystal was glued using gold paste to a gold wire and served as a working electrode. Electrochemical etching reactions were carried out using cyclic voltammetry from 0.05 to –0.35 V versus RHE with a scan rate of 10 mV s^–1^ in 1 M HClO_4_ (pH = 0) at different temperatures using an Ag/AgCl reference electrode saturated with KCl and a Pt plate as a counter electrode, where the temperature was set at 25, 40, 55 and 65°C with 50 cycles. Both the anode and cathode electrodes of the PEM electrolyser were prepared using electrochemically etched PdCoO_2_ powder glued using gold paste onto 0.5 cm^2^ of titanium plates, where the mass loading of the Pd catalyst was 2 mg cm^–2^. A commercial IrO_2_ and Pd/C catalyst (10 wt%, Pd nanoparticles supported on activated charcoal) were used as benchmarks. The catalyst inks for Pd/C and IrO_2_ samples were prepared by mixing 10 mg of catalysts with 10 mg of carbon black (XC-72) in 750 μL isopropanol, 250 μL of H_2_O and 60 μL of 5% Nafion solution followed by ultrasonication for 90 min, while no extra carbon black was added in the Pd/C dispersion; 7 μL of the homogeneous catalyst ink was loaded onto a glassy carbon electrode with 3-mm diameter.

### Characterization

A Philips X’Pert Pro Super diffractometer was utilized to detect X-ray powder diffraction (XRD), with a wavelength of 1.54178 Å for Cu-Kα radiation. SEM was performed using a JEOL JSM-6700F SEM. TEM was performed using a Hitachi H-7700 operated at 100 kV. A JEOL JEM-ARF200F TEM/STEM was used to execute high-angle annular dark-field scanning transmission electron microscopy (HAADF-STEM) and corresponding energy-dispersive X-ray spectroscopy mapping. The X-ray photoelectron spectra were carried out using a Thermo ESCALAB250Xi spectrometer (monochromatized Al Kα as the excitation source, hν = 1486.6 eV) and a pass energy of 30 eV, with the binding energies being calibrated using the C 1-s peak of contaminant carbon at 284.80 eV. The dissolution of Pd in durability was recorded with an inductively coupled plasma–atomic emission spectrometer using an Optima 7300 DV (PerkinElmer Corporation).

## Supplementary Material

nwac108_Supplemental_File
