# Supplementary material for: Hierarchical palladium catalyst for highly active and stable water oxidation in acidic media
Source: Natl Sci Rev. 2022 Jun 9;10(2):nwac108. doi: 10.1093/nsr/nwac108 (PMC10035503; doi:10.1093/nsr/nwac108)
Supplement: nwac108_Supplemental_File [file nwac108_supplemental_file.pdf]

Supporting information

Hierarchical Palladium Catalyst for Highly Active and Stable Water Oxidation in Acidic Media

Jing Peng,[†1] Haofeng Sun,[†1] Kun Ni,[†1] Jiajing Wu,[1] Xinyu Sun,[1] Yueqi Su,[1] Han Cheng,[1] Yuhua Liu,[1] Yuqiao Guo,[1] Wentuan Bi,[12] Yanwu Zhu,[1] Changzheng Wu,[12]* and Yi Xie[12]

1 Hefei National Laboratory for Physical Science at the Microscale, CAS Center for Excellence in Nanoscience, iChEM (Collaborative Innovation Center of Chemistry for Energy Materials), and CAS Key Laboratory of Mechanical Behavior and Design of Materials, University of Science and Technology of China, Hefei, Anhui, 230026, P. R. China

2 Institute of Energy, Hefei Comprehensive National Science Center, Hefei, Anhui, 230026, P. R. China.

†These authors contributed equally to this work.

*Correspondence: czwu@ustc.edu.cn.

Electrochemical measurements.

The electrocatalytic measurements were performed in a three-electrode system on an electrochemical workstation (CHI760E). As-prepared Pd catalysts were served as working electrodes and performed by linear sweep voltammetry (LSV) tests with a scan rate of 5 mV s^{-1} at 25 ℃. All of the potentials were calibrated to the reversible hydrogen electrode (RHE) by Nernst equation. Electrochemical impedance spectroscopy (EIS) measurements were recorded at different potentials versus Ag/AgCl with 5 mV amplitude in a frequency range from 100 kHz to 0.1 Hz. To account for the iR drop, the anode polarization curves were corrected by subtracting i × R (current multiplied by series resistance). The durability tests were carried out by chronopotentiometric measurements in 1 M $HClO_4$.

For each catalyst, the mass activity was obtained by normalizing current density to the corresponding mass loaded density of Pd network (M_{Pd}). M_{Pd} were calculated by measuring thickness of Pd network through SEM. And thus M_{Pd} can be given from the following calculation

$$M_{Pd} = D \times \rho\ (PdCoO_2) \times wt\%\ (Pd),$$

Where D is the thickness of Pd capping, and ρ ($PdCoO_2$) is the mass density of $PdCoO_2$ (7.99 g cm^{-3}).[1] Owing to the porosity of Pd-network, the Pd content of Pd-55 capping layer was slightly overrated. The ECSAs of palladium catalysts were measured from charges associated with underpotentially deposited Hydrogen (H_{upd}) and stripping of CO.[2] For H_{upd}, CVs were recorded in 1 M $HClO_4$ with a scan rate of 50 mV s^{-1}. The H_{upd} peaks were used to calculate ECSAs by assuming a charge density of 240 μC cm^{-2}, which is a value for one monolayer of hydrogen coverage on Pd (111) surface. This method inevitably overrated the ECSAs resulting from the incorporation of hydrogen in both surface and inner of Pd. Therefore, CO stripping is adopted to accurately measure the ECSA of hierarchical Pd catalysts. For the CO stripping experiments, CO gas was first bubbled into 1 M $HClO_4$ while holding the working electrode potential at 0.1 V versus RHE for 20 min. After purging the above electrolyte with N_2 for at least 30 min, two CVs were recorded at a scan rate of 20 mV s^{-1}. The ECSAs were calculated from the charge of CO stripping (the first CV) by subtracting the background charge

(the second CV) and then normalized by M_{Pd}, assuming a charge density of 420 μC cm^{-2}.

As for PEM electrolyzer measurements, the as-prepared Pd electrodes were performed as both anode and cathode in the homemade electrolyzer devices. Then the electrolyzer was characterized by stepping the anode current density from 30 mA cm^{-2} to 2.5 A cm^{-2} in 1 M $HClO_4$ at 65 ℃. Corresponding potentials were reasonably iR corrected.

Simulation method.

The OER reaction is considered as four-electron process. The calculations were done by using the Vienna ab initio simulation package (VASP). The exchange correlation interactions were described by generalized gradient approximation (GGA) with Perdew−Burke−Ernzerhof (PBE) functional. The energy cutoff of plane wave basis set was 400 eV. The spin of electrons was unrestricted. Gaussian type smearing was used with energy window of 0.05 eV. The energy convergence tolerance for the self-consistent field (SCF) was 0.1 meV and the force convergence tolerance for geometry optimization was 0.02 eV/Å. A 2 × 2 × 1 super cell model of Pd (111) surface is considered, containing 64 Pd atoms and 4 Pd atom layers. 1 × 1 × 1 k points sampling was used for geometry optimizations and 2 × 2 × 1 Gamma centered k points sampling was used for single energy calculations of the surface adsorption models. Grimme DFT-D3 method was used to describe long-range van der Waals (vdW) interactions with B-J damping. The lower two layers of the four Pd layers in the surface model is fixed during geometry optimization. After testing, the ZPE and TS term of the strained models in Gibbs free energy calculation is kept consistent with the unstrained adsorption models to reduce the calculation cost, with introducing ~0.03 eV errors in final ΔG values.

S1. HER process for $PdCoO_2$.

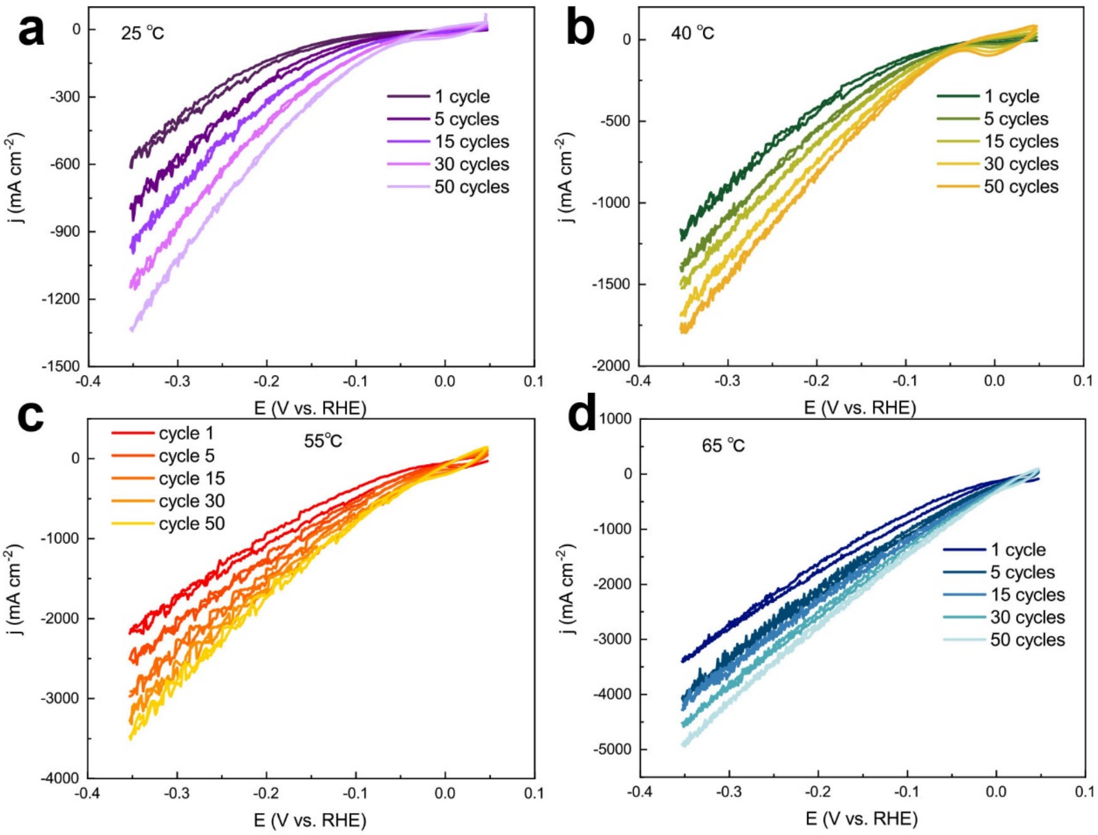

Figure S1. (a-d) HER process for $PdCoO_2$ at 25 ℃, 40 ℃, 55 ℃ and 65 ℃ in 1 M $HClO_4$ during cathodic cycling with a scan rate of 10 mV s^{-1} (without iR corrected).

Similar to previous reports,[3, 4] the HER performance of $PdCoO_2$ can be greatly improved by cycling because of the formation of Pd capping. Prominently, the cycle-dependent currents increased much more rapidly when treated at higher working temperature.

S2. Structural evolution of $PdCoO_2$.

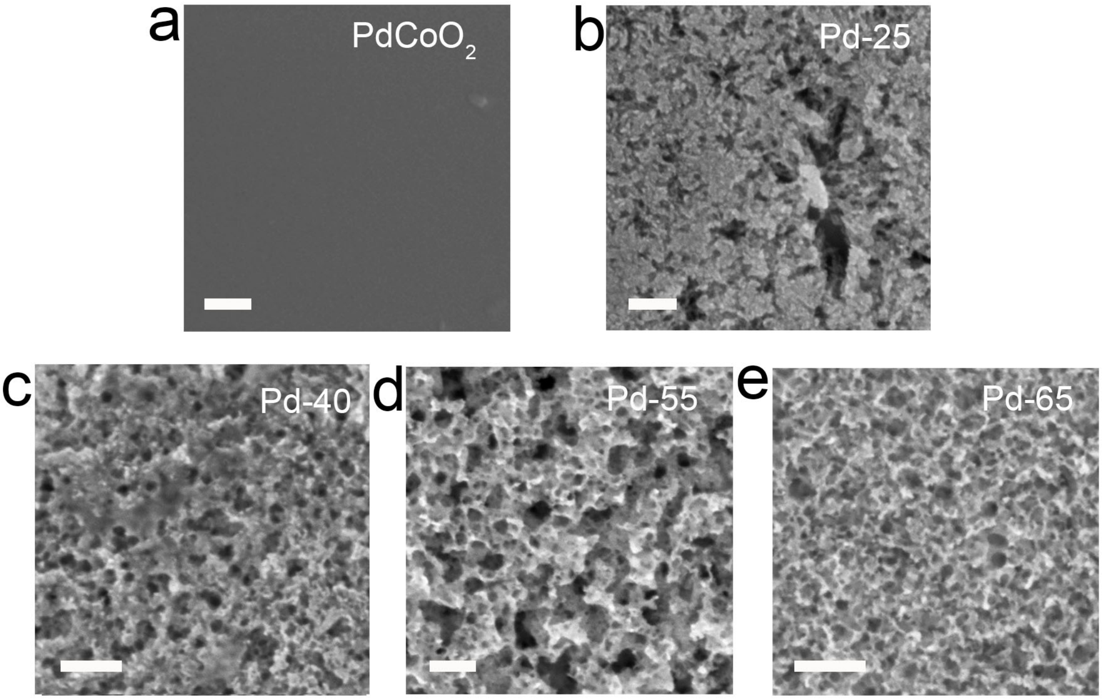

Figure S2. Structural evolution of $PdCoO_2$ after thermal electrochemical etching process at different temperatures. (a-e) SEM image of $PdCoO_2$ single crystal, Pd-25, Pd-40, Pd-55 and Pd-65, respectively.The scale bars are 100 nm.

S3. HAADF image of Pd-55 cross-section.

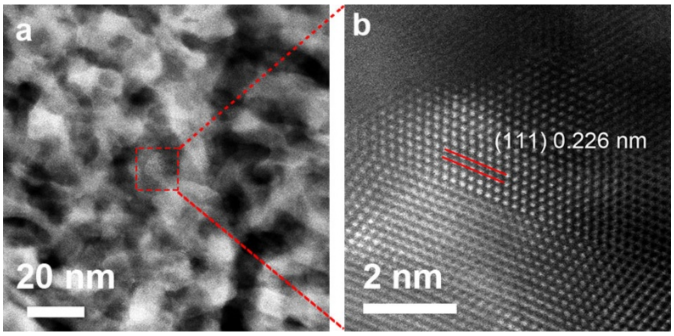

Figure S3. Cross-section HAADF-STEM images of Pd-55 frameworks. (a)Pd-55 frameworks and (b) its atomic-resolution image.

Cross-section HAADF-STEM images of Pd-55 reveals a porous network with the diameter of the skeletons less than 20 nm. The lattice spacing distance of 2.26 Å is in accordance with the Pd (111) facet.

S4. STEM image of hierarchical Pd-55 catalysts.

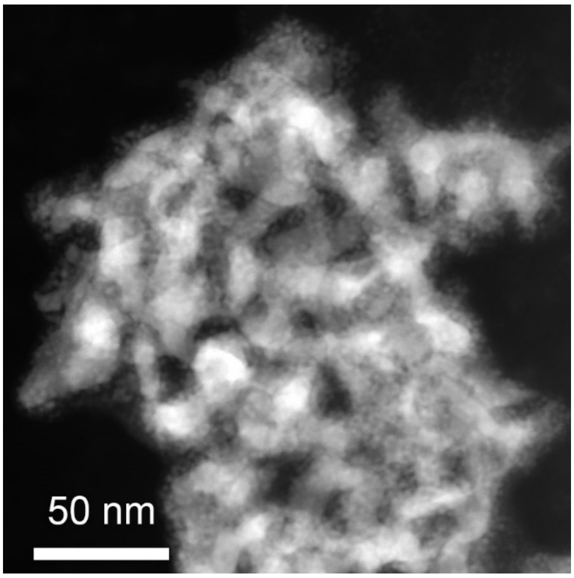

Figure S4. STEM image of hierarchical Pd-55 catalysts with Pd NPs anchored on porous Pd framework.

Hierarchical structure can be observed directly in **Figure S4** with porous Pd networks anchored by lots of small-sized Pd nanoparticles.

S5. XPS spectra of Pd catalysts.

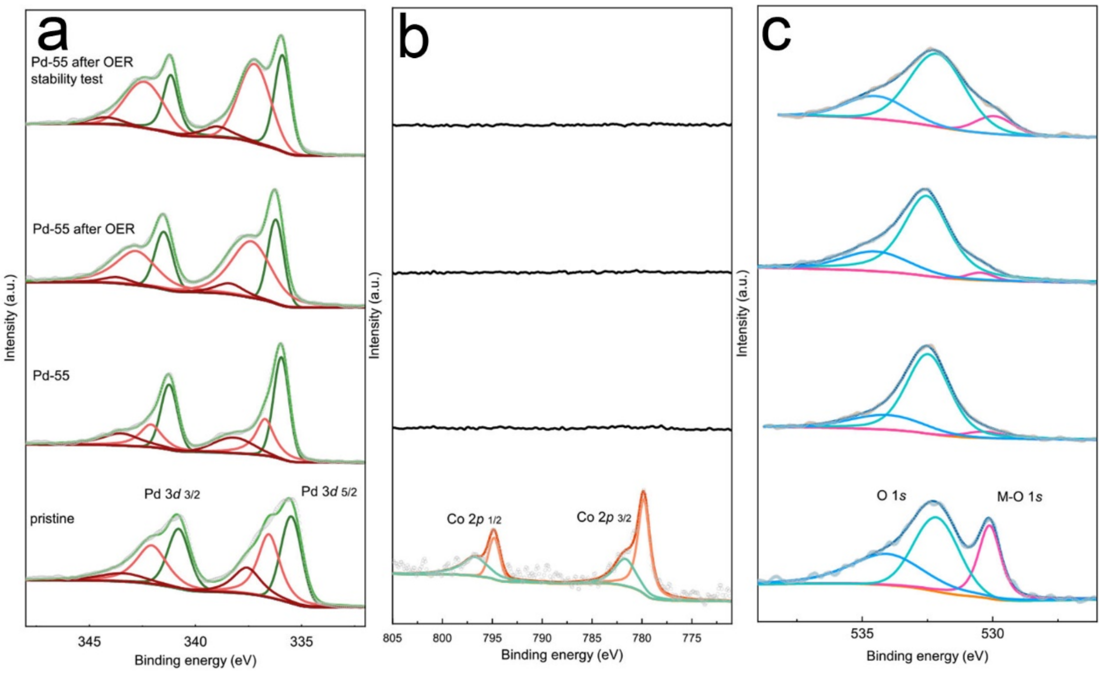

Figure S5. XPS spectra of Pd catalysts. (a-c) Pd 3d, Co 2p and O 1s peaks of pristine $PdCoO_2$, Pd-55, Pd-55 after OER and OER durability test in 1M $HClO_4$, respectively.

After thermal electrochemical etching process, the Co and lattice oxygen are entirely eroded, which can be confirmed by the XPS analysis in **Figure S5**. Compared to the as-prepared Pd-55, high oxidation state at 336.6 eV increases significantly when OER test carried out, indicating the formation of PdO_x species on Pd surfaces during OER process.

S6. H_{upd} measurements of Pd catalysts.

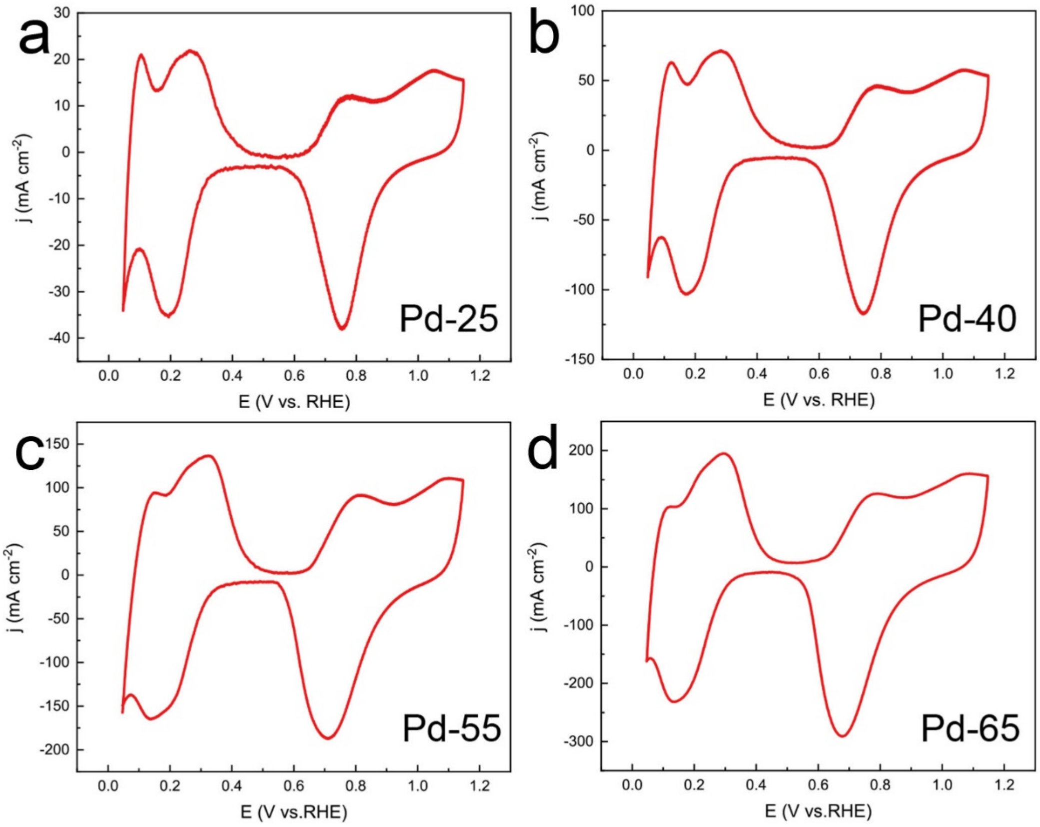

Figure S6. H_{upd} measurements of Pd catalysts. (a-d) CVs of Pd-25, Pd-40, Pd-55 and Pd-65catalysts in 1 M $HClO_4$ with a scan rate of 50 $mV \cdot s^{-1}$, respectively.

There are two desorption of H_{upd} peaks for Pd catalysts between 0~0.4 V. The peak at ~0.26 V corresponds to the hydrogen adsorption on Pd surface (forming Pd-H), while that at ~0.11 V indicates the incorporation of hydrogen inner Pd, forming PdH_x.[3] The calculated ECSAs were 310.5 m^2 g^{-1} for Pd-25, 645.5 m^2 g^{-1} for Pd-40, 702.3 m^2 g^{-1} for Pd-55 and 472.2 m^2 g^{-1} for Pd-65, respectively, which could be much overestimated on account of involving PdH_x peaks.

S7. CO stripping voltammograms for Pd catalysts.

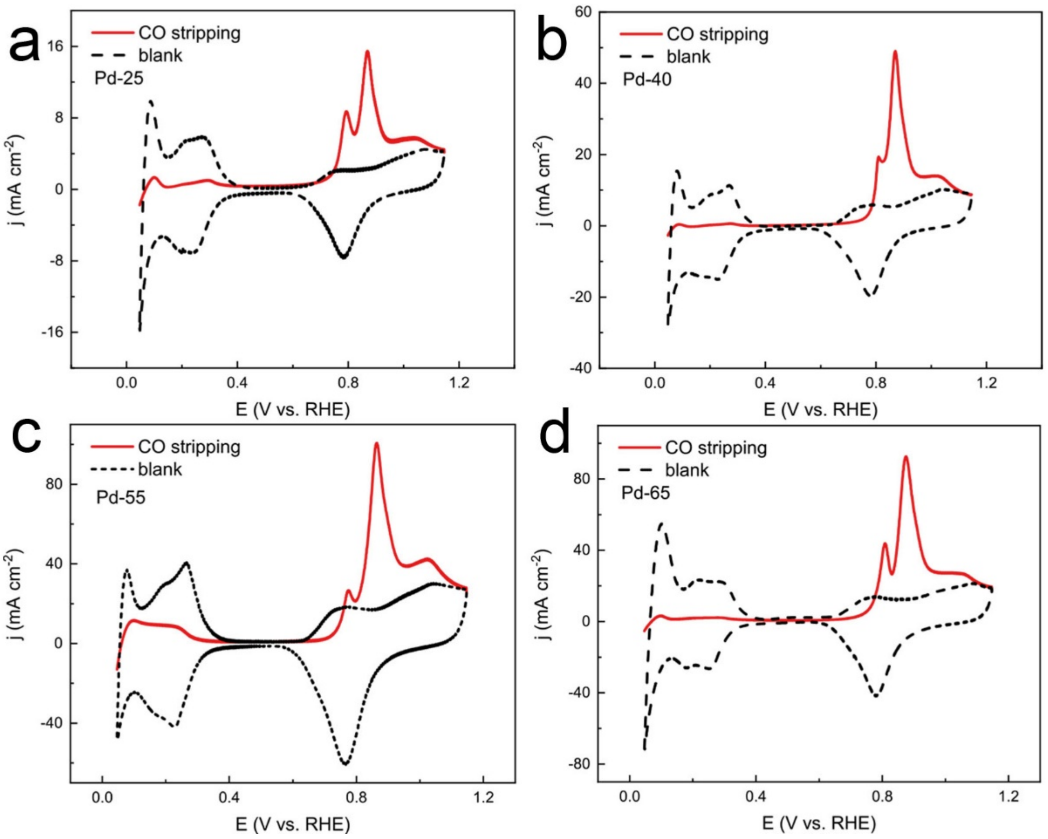

Figure S7. CO stripping voltammograms for Pd catalysts. (a-d) CVs of Pd-25, Pd-40, Pd-55 and Pd-65 catalysts, respectively.

The integrated charge of CO stripping is used to calculate the ECSA of Pd catalysts. From Figure S5a-5c, Pd catalysts exhibit a gradual enlarging current density, which can be ascribed to the increasing porosity of hierarchical Pd. However, the ECSAs normalized by Pd loaded mass (detailed in supporting method) display slightly difference (shown in **Figure 2g**) as against large variations of current density, since the thickness of Pd capping can also be increased as the temperature elevated.

S8. Thickness measurement of Pd-55 capping layers by SEM.

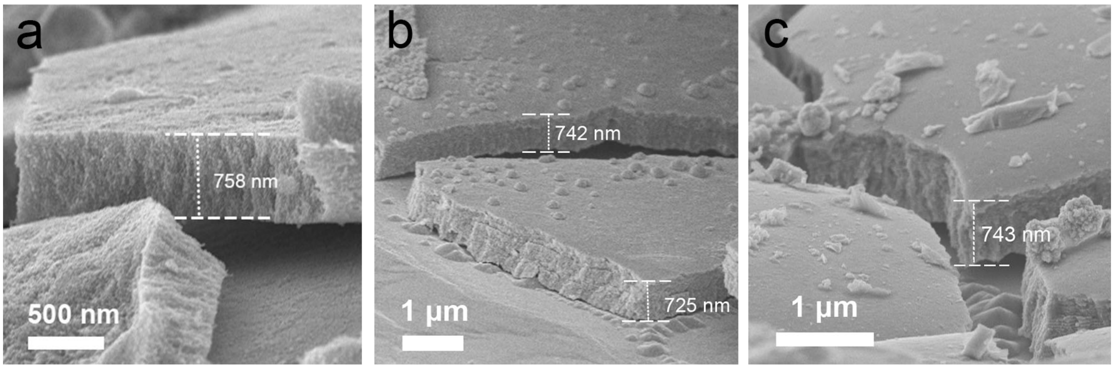

Figure S8. (a-c) SEM images of Pd-55 frameworks adhering to the underlying $PdCoO_2$ on three different samples.

To estimate the thickness of Pd-55 frameworks, Pd-55 samples were prepared and characterized by SEM. As shown in **Figure S8**, these porous Pd slabs still attaching to the underlying $PdCoO_2$, and the side views of them give an average thickness of ~740 nm.

S9. OER LSV curves of Au wire, $PdCoO_2$ and Pd-65.

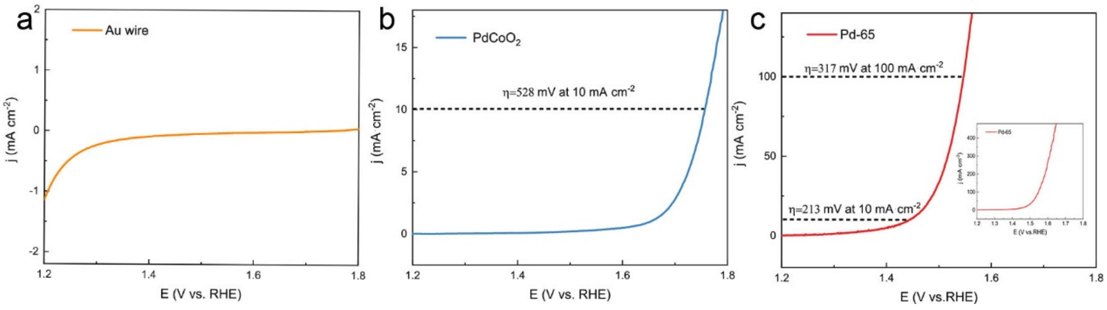

Figure S9 (a-c) LSV curves of Au wire, $PdCoO_2$ and Pd-65 in 1 M $HClO_4$ at 25 ℃ with a scan rate of 5 mV s^{-1}, respectively.

Au displayed extremely poor OER performance and ignorant contribution in electrochemical measurement. Fresh $PdCoO_2$ crystals had inferior OER catalytic performance, the overpotential of which to reach the current density of 10 mA cm^{-2} was 528 mV. As mentioned in **Figure 3a**, dramatically increase of OER activity were achieved after electrochemical corrosion. When compared to Pd-55, Pd-65 performed a similar OER curve, while the overpotential of the latter is larger than the former.

S10. Nyquist plots for Pd-25, Pd-40 and Pd-55.

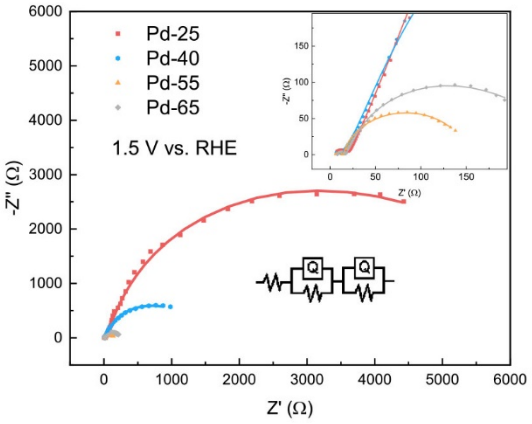

Figure S10. Nyquist plots for Pd-25, Pd-40 and Pd-55 studied by electrochemical impedance spectroscopy (EIS) at 1.5 V_{RHE} in 1 M $HClO_4$.

S11. OER performance of Pd catalysts at high current density and high overpotentials.

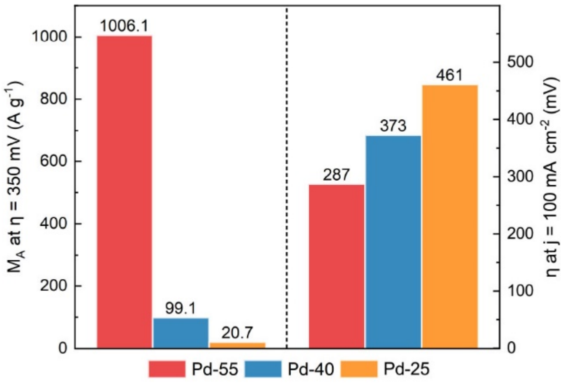

Figure S11. Mass activity at η = 350 mV (left) and Overpotential at j = 100 mA cm^{-2}(right) for the Pd catalysts.

The reactivities at large current density and high overpotential are essential for practical applications. Therefor **Figure S11** displays mass activities at 350 mV and overpotentials at 100 mA cm^{-2} of Pd catalysts. The Pd-55 still possesses the best reactivity for highest mass activity of 1006.1 A g^{-1} and the lowest overpotential of 287 mV to reach 100 mA cm^{-2}.

S12. HER process of $PdCoO_2$ for 500 cycles.

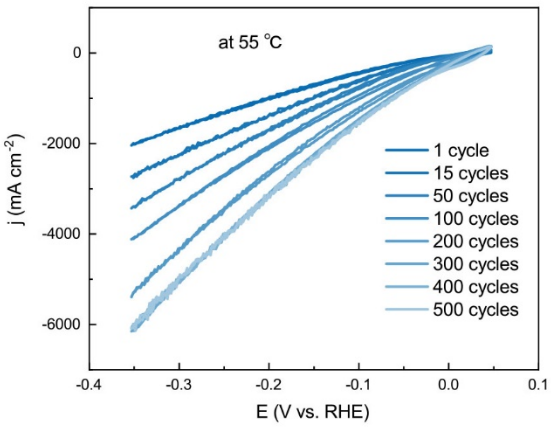

Figure S12.HER process for $PdCoO_2$ with 10 mV s^{-1} scan rate and 500 cycles from -0.35 to 0.05 V_{RHE} in 1 M $HClO_4$ at 55 ℃ (without iR corrected)..

S13. TEM image and electrochemical performance of synthesized Pd NPs.

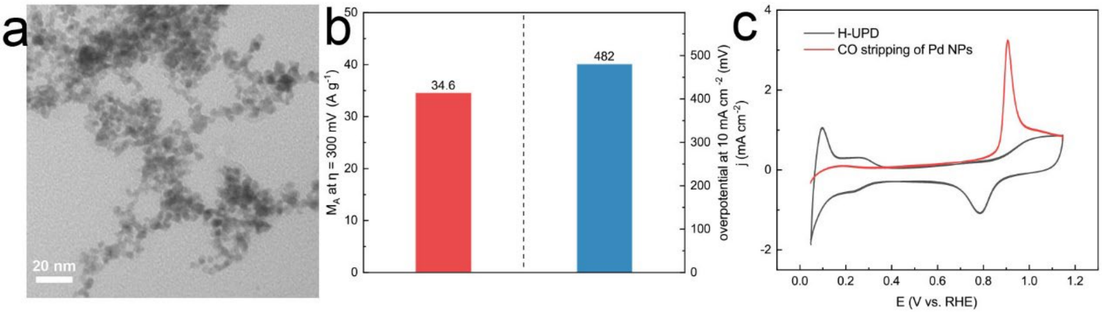

Figure S13. Characterizations of synthesized Pd NPs: (a) TEM image, (b) mass activity and overpotential, (c) CO stripping voltammogram.

To further investigate the size-effect, Pd NPs with average size of 5.4 nm were synthesized and measured the OER performance. The mass activity of 34.6 A g^{-1} at 300 mV overpotential is comparable with Pd-40, which has similar Pd particle size for ~ 6 nm. However, its low ECSA of 21.2 m^2 g^{-1} ascribed to the absence of Pd frameworks makes the Pd NPs show a much higher overpotential of 482 mV than 280 mV of Pd-40.

S14. SEM of Pd-55 surface after stability test.

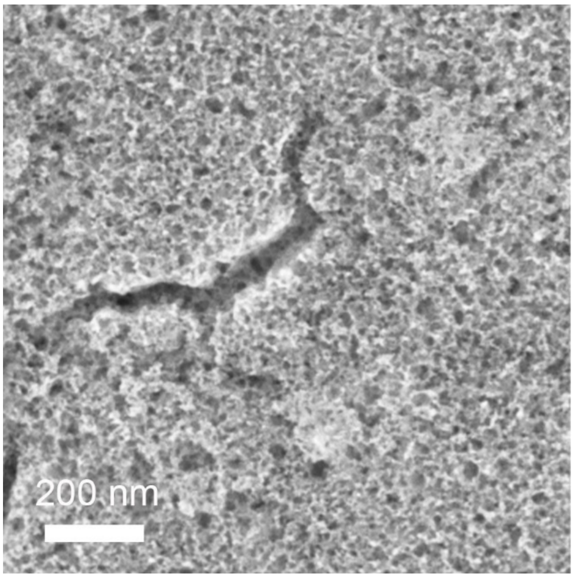

Figure S14.SEM image of Pd-55 surface after 500-hour stability test. The maintained porosity indicates high stability of Pd-55 frameworks.

S15. OER pathway of Pd within acidic condition.

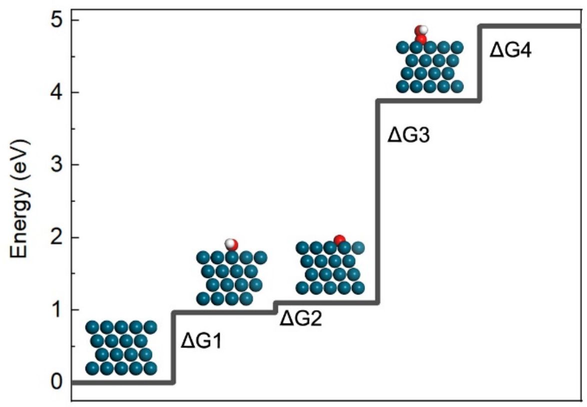

Figure S15. OER pathway of Pd within acidic condition.

S16. SEM and TEM and OER performance of Pd-55 film.

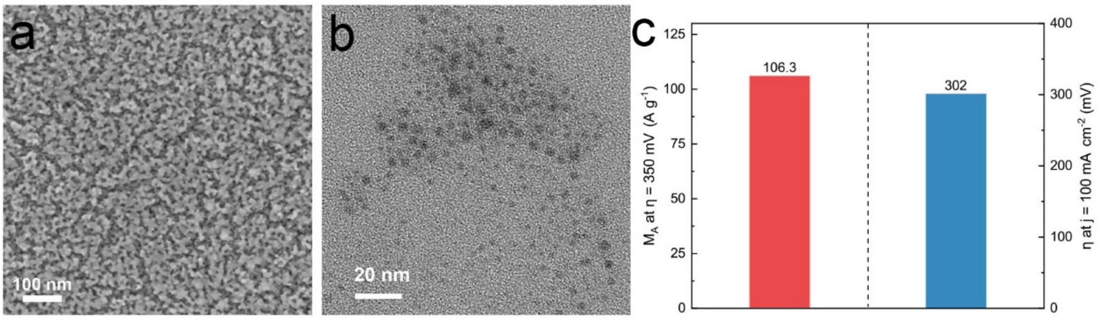

Figure S16.SEM (a) and TEM (b) images of Pd-55 film. (c) OER performance of Pd-55 film with a three-electrode system.

In **Figure S16**, Pd-55 film has hierarchical structure as well with porous frameworks and ~4.6 nm-sized Pd NPs. OER performance test of Pd-55 films was carried out with a three-electrode system in 1 M $HClO_4$ at room temperature. The mass activity of 106.3 A g^{-1} at 350 mV overpotential is much smaller than 1006.1 A g^{-1} of Pd-55 prepared with single crystals (**Figure S11**), which could be contributed from the larger-sized Pd NPs. Nonetheless, the film displays similar overpotential of 302 mV to reach 100 mA cm^{-2} as a result of the high mass loading.

S17. LSV of water electrolyzer with pure Ti plates as working electrodes.

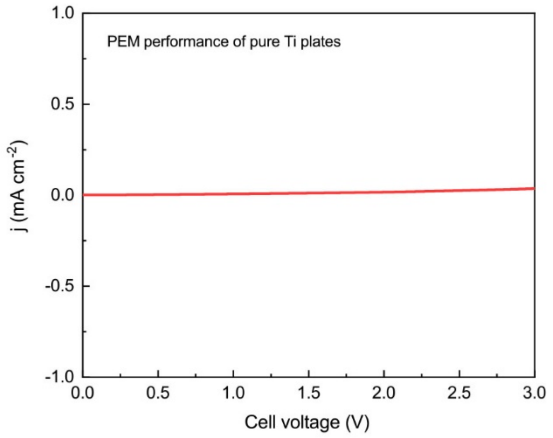

Figure S17. PEM performance using pure Ti plated as both cathode and anode. The test was carried in 1 M $HClO_4$ at 65 ℃ with a scan rate of 5 mV s^{-1}.

Table S1. OER performance of Pd catalysts prepared with different scan cycles and scan rates.

Table S1. OER performance of Pd catalysts prepared at 55 ℃ with different scan cycles and scan rates.

Factors in electrochemical cycling process		Overpotential at 10 mA cm^{-2} (mV)	Mass activity at 300 mV overpotential (A g^{-1})	Size of Pd NPs (nm)
Cycle numbers at 10 mV s^{-1}	25	266	88.6	3.7
	50	197	407.2	2.8
	100	243	62	4.1
	500	201	52.38	4.2
Scan rates with 50 cycles	5	204	314.6	3.1
	10	197	407	2.8
	20	274	91.4	3.8
	30	242	234.2	3.4

Investigations on the impacts of scan cycles and scan rates were also carried out, all Pd catalysts were prepared with scan range from -0.35 to 0.05 V_{RHE} in 1M $HClO_4$ at 55 °C. As summarized in **Table S1**, the sample prepared with 10 mV s^{-1} and 50 cycles displays the lowest overpotential for 197 mV and highest mass activity for 407.2 A g^{-1} at η = 300 mV, which can be resulted from the size-effect as demonstrated in main text. It is worth nothing that the HER current can be saturated after 300 cycles (**Figure S12**), and the ~4.2 nm-size Pd NPs after 500 cycles hinder its mass activity of 52.38 A g^{-1}. However, the high mass loading of Pd catalysts, 2.56 mg cm^{-2}, compensates its reactivity and displays a low overpotential of 201 mV.

Table S2. Acidic OER performance Pd-55 with Ir- and Ru- based catalysts.

Table S2. Acidic OER performance Pd-55 with Ir- and Ru- based catalysts.

Catalyst	Electrolyte	η at 10 mA cm^{-2} (mV)	Mass activity	Tafel slope (mV dec^{-1})	Degradation rate (μV h^{-1})	Reference
Pd-55	1 M $HClO_4$	197	407.2 A g^{-1} at 1.53 V	51.3	66	This work
Mesoporous Ir	0.5 M H_2SO_4	240	260 A g^{-1} at 1.5 V	49	6250	5
$Ir_{44}Pd_{10}$ nanocages	0.1 M $HClO_4$	224	1990 A g_{Ir}^{-1} at 1.48 V	53.9	600	6
$W_{0.2}Er_{0.1}Ru_{0.7}O_{2-\delta}$	0.5 M H_2SO_4	167	1518.6 A g^{-1} at 1.505 V	66.8	166	7
IrO_2 nanoneedles	1 M H_2SO_4	313	56.1 A g^{-1} at 1.55 V	57	15000	8
IrO2@Ir/TiN	0.5 M H_2SO_4	265	480.4 A g_{Ir}^{-1} at 1.6 V	52.3	2933	9
RuO_2 nanosheets	0.1 M $HClO_4$	255	56.1 A g^{-1} at 1.48 V	38	43300	10
RuO_2/carbon clothes	0.5 M H_2SO_4	179	\	36.9	950	11
Cu-RuO_2	0.5 M H_2SO_4	188	\	43.96	10375	12
Co-RuIr nanocrystals	0.1 M $HClO_4$	235	\	66.9	5800	13
$Ru_1Ir_1O_x$	0.5 M H_2SO_4	204	1224.6 A $g_{noble\ metal}^{-1}$ at 1.53 V	71.3	270	14
$RuNi_2$©G-250	0.5 M H_2SO_4	227	57.6 A g_{Ru}^{-1} at 1.48 V	65	1000	15

References

1. Shannon RD, Prewitt CT, Rogers DB. Chemistry of noble metal oxides. II. Crystal structures of platinum cobalt dioxide, palladium cobalt dioxide, coppper iron dioxide, and silver iron dioxide. *Inorg Chem*. 1971; **10**(4): 719-23.
2. Luo M, Zhao Z, Zhang Y*, et al.* PdMo bimetallene for oxygen reduction catalysis. *Nature*. 2019; **574**(7776): 81-5.
3. Li G, Khim S, Chang CS*, et al.* In Situ Modification of a Delafossite-Type $PdCoO_2$ Bulk Single Crystal for Reversible Hydrogen Sorption and Fast Hydrogen Evolution. *ACS Energy Lett*. 2019; **4**(9): 2185-91.
4. Podjaski F, Weber D, Zhang S*, et al.* Rational strain engineering in delafossite oxides for highly efficient hydrogen evolution catalysis in acidic media. *Nat Catal*. 2019; **3**(1): 55-63.
5. Jiang B, Guo Y, Kim J*, et al.* Mesoporous Metallic Iridium Nanosheets. *J Am Chem Soc*. 2018; **140**(39): 12434-41.
6. Zhu J, Chen Z, Xie M*, et al.* Iridium-Based Cubic Nanocages with 1.1-nm-Thick Walls: A Highly Efficient and Durable Electrocatalyst for Water Oxidation in an Acidic Medium. *Angew Chem Int Ed Engl*. 2019; **58**(22): 7244-8.
7. Hao S, Liu M, Pan J*, et al.* Dopants fixation of Ruthenium for boosting acidic oxygen evolution stability and activity. *Nat Commun*. 2020; **11**(1): 5368.
8. Lim J, Park D, Jeon SS*, et al.* Ultrathin IrO_2 Nanoneedles for Electrochemical Water Oxidation. *Adv Funct Mater*. 2018; **28**(4): 1704796.
9. Li G, Li K, Yang L*, et al.* Boosted Performance of Ir Species by Employing TiN as the Support toward Oxygen Evolution Reaction. *ACS Appl Mater Interfaces*. 2018; **10**(44): 38117-24.
10. Laha S, Lee Y, Podjaski F*, et al.* Ruthenium Oxide Nanosheets for Enhanced Oxygen Evolution Catalysis in Acidic Medium. *Adv Energy Mater*. 2019; **9**(15): 1803795.
11. Ge RX, Li L, Su JW*, et al.* Ultrafine Defective RuO_2 Electrocatayst Integrated on Carbon Cloth for Robust Water Oxidation in Acidic Media. *Adv Energy Mater*. 2019;

9(35): 1901313.
12. Su J, Ge R, Jiang K, *et al.* Assembling Ultrasmall Copper-Doped Ruthenium Oxide Nanocrystals into Hollow Porous Polyhedra: Highly Robust Electrocatalysts for Oxygen Evolution in Acidic Media. *Adv Mater*. 2018; **30**(29): e1801351.
13. Shan J, Ling T, Davey K, *et al.* Transition-Metal-Doped RuIr Bifunctional Nanocrystals for Overall Water Splitting in Acidic Environments. *Adv Mater*. 2019; **31**(17): e1900510.
14. He J, Zhou X, Xu P, *et al.* Regulating Electron Redistribution of Intermetallic Iridium Oxide by Incorporating Ru for Efficient Acidic Water Oxidation. *Angew Chem Int Ed Engl*. 2021; **11**(48): 2102883.
15. Cui X, Ren P, Ma C, *et al.* Robust Interface Ru Centers for High-Performance Acidic Oxygen Evolution. *Adv Mater*. 2020; **32**(25): e1908126.
